# WDR4-mediate tRNA m7G modification to promote mitophagy and browning of white adipose tissue for ameliorating obesity in male mice

**DOI:** 10.1080/21623945.2025.2588888

**Published:** 2025-11-25

**Authors:** Beisi Lin, Chaofan Wang, Yanling Yang, Zhigu Liu, Panwei Mu, Wen Xu, Yonghui Li

**Affiliations:** a Department of Endocrinology & Metabolism, The Third Affiliated Hospital of Sun Yat-Sen University, Guangzhou, China; b Division of Cardiovascular Surgery, The Sun Yat-Sen Memorial Hospital of Sun Yat-Sen University, Guangzhou, China

**Keywords:** Obesity, tRNA m7G modification, mitophagy, brown adipose tissue, WDR4

## Abstract

Objective: Brown adipose tissue activation is a potential anti-obesity strategy. N^7^-methylguanosine (m^7^G) modification is a novel RNA epigenetic modification, but its role in adipose metabolism remains unexplored. Methods: Male mice were fed a high-fat diet (HFD), followed by PCR array screening. Gain-of-function experiments and TRAC-seq were employed to explore WDR4 function. Result: A Mouse Epigenetic Modification Enzymes PCR Array revealed that WDR4 expression showed the most pronounced downregulation in HFD mice. Overexpression of WDR4 in 3T3-L1 cells and primary adipocytes significantly increased UCP1 expression and suppressed lipid droplet formation, and enhanced mitophagy as evidenced by mitochondrial ultrastructure, autophagic vesicles, and LC3 expression. Suppression of mitophagy using 3-MA and bafilomycin A1 attenuated WDR4-induced adipocyte browning. WDR4 overexpression enhanced translational activity and reshaped the tRNA m^7^G methylome in 3T3-L1 adipocytes, specifically induced 38 unique tRNA m^7^G modification sites, and increasing cleavage scores of multiple tRNAs. GSE229240 dataset revealed that WDR4 mutation significantly reduced translation efficiency of 195 genes enriched in the TGF-β signalling , including BMP8B. Knockdown of BMP8B partially counteracted WDR4-mediated mitophagy. Conclusion: WDR4 promotes adipocyte browning by enhancing BMP8B translation through tRNA m^7^G modification, revealing a novel m^7^G epitranscriptomic mechanism with therapeutic potential for obesity.

## Introduction

Obesity, as a global health challenge, has been increasing at an alarming rate, leading to numerous metabolic disorders and posing a severe threat to human health [1]. Recent studies indicate that the prevalence of obesity among children and adolescents in China has reached 7.77% [2]. White adipocytes, when exposed to certain stimuli (such as cold exposure, β-adrenergic receptor agonist treatment, or intense exercise), undergo a process known as ‘browning’, this process involves the upregulation of mitochondrial brown fat uncoupling protein 1 (UCP1) expression and the enhancement of mitochondrial quantity and function, thereby making them more similar to brown adipocytes [3]. The browning process of adipocytes plays a crucial role in combating obesity by enhancing energy expenditure and thermogenesis, thereby reducing fat accumulation [4]. Activating or increasing the quantity and functionality of brown adipose tissue is of great significance in elevating basal metabolic rate and improving obesity [5]. Therefore, an in-depth investigation into the molecular mechanisms underlying BAT activation is essential for developing novel therapeutic strategies for obesity, offering new hope for patients and alleviating the health burden of obesity on both individuals and society.

RNA epigenetic modifications, which target RNA molecules, play a pivotal role in various physiological and pathological processes [6]. Zhang et al. observed differences in the methylation levels and methylated transcripts in BAT of mice following thermoneutral or cold stimulation [7]. Wu et al. demonstrated that the m5C methylation-binding protein YBX1 acts as a transcription factor to promote the transcription and expression of Ulk2, enhancing autophagy and adipogenesis [8]. Tao et al. revealed that the PGE2-EP3 signalling axis enhances the stability of Zfp410 mRNA through WTAP-mediated m6A modification, facilitating the differentiation of human embryonic stem cells into mature brown adipocytes [9]. These findings underscore the significance of RNA modifications in the regulation of adipogenesis. It is noteworthy that our previous findings revealed the involvement of acetylation modification in the browning of white adipocytes. Specifically, GLP-1 R agonists promote the browning remodelling of white adipose tissue by enhancing mitochondrial biogenesis and function in a SIRT1-dependent manner [10]. Moreover, m^7^G modification, characterized by methylation at the seventh nitrogen position of guanosine, represents a pivotal post-transcriptional RNA modification that dynamically regulates mRNA stability, splicing, and translational efficiency across diverse biological processes [11]. WDR4 serves as an essential cofactor for METTL1, facilitating the deposition of m7G modifications at position 46 in the variable loop of tRNAs, which plays a pivotal role in maintaining tRNA structural stability and functional integrity [12]. However, the mechanisms underlying the role of WDR4-mediated m^7^G epigenetic modification changes in adipocyte browning remain to be further elucidated.

Mitophagy, a key pathway for maintaining mitochondrial homoeostasis and cellular energy metabolism, functions by precisely identifying and eliminating damaged or dysfunctional mitochondria [13]. Mitophagy is primarily achieved through PINK1-Parkin-dependent and independent pathways: on damaged mitochondria, PINK1 is stabilized and activated, subsequently recruiting Parkin, which mediates the ubiquitination of mitochondrial outer membrane proteins, promoting the formation of autophagosomes that engulf the mitochondria, ultimately leading to their degradation via fusion with lysosomes [14]. Mitophagy is essential for mitochondrial quality control and metabolic homoeostasis. Recent studies have reported that mitophagy-mediated mitochondrial homoeostasis plays a crucial role in regulating the browning of white adipose tissue [15]. For instance, FUNDC1 deficiency impairs mitochondrial quality and exacerbates diet-induced obesity and metabolic syndrome [16], Parkin regulates obesity by coordinating mitophagy [17], and YBX1 promotes brown adipogenesis and thermogenesis through mitophagy [18]. Conversely, inhibition of autophagy induces obesity, as demonstrated by the adipokine asprosin secreted by white adipose tissue, which inhibits adipocyte mitophagy, prevents white adipose tissue browning in mice, and promotes obesity [19]. Therefore, mitophagy plays a pivotal role in adipocyte browning. However, whether epigenetic modifications can synergize with mitophagy to influence adipocyte browning remains unclear.

This study aims to investigate the molecular mechanisms by which specific epigenetic- modifying enzymes regulate adipocyte browning through mitophagy. Initially, we identified the key molecule WDR4 using an epigenetic-modifying enzyme PCR array. Subsequently, gain-of-function experiments demonstrated that WDR4 mediates adipocyte browning by regulating mitophagy. Finally, the downstream tRNA-modified landscape by WDR4 was screened and analysed using Trac-seq. Our findings not only reveal the critical role of WDR4 in adipocyte browning but also provide novel insights for understanding obesity and related metabolic disorders.

## Materials and methods

### Mice (only male) treatment

All animal experiments were approved by the Animal Ethics Committee of The Third Affiliated Hospital of Sun Yat-sen University. Ten 7-week-old male C57BL/6J mice (purchased from Beijing SPF Biotechnology Co., Ltd.) were housed under controlled conditions with a temperature of 24–26°C, humidity of 55–60%, and a 12-hour light/dark cycle, with free access to water. Prior to the experiment, all mice underwent a 7-day acclimatization period. After the acclimatization period, the mice were randomly assigned to the normal chow diet (NCD) group or high-fat diet (HFD) group, with 5 mice in each group, using a random number table. Starting from the 8th week of age, the HFD group was fed a high-fat diet [10] (Basic feed +10% lard +10% sucrose +10% egg yolk powder +1.2% cholesterol +0.2% sodium cholate), while the NCD group continued to receive a normal diet. Body weight and food intake were recorded weekly. After 8 weeks of intervention (i.e. at 16 weeks of age), all mice were fasted for 12 hours, anesthetized with CO_2_, and killed by cervical dislocation. Epididymal adipose tissue was collected for subsequent analysis.

### Immunohistochemical (IHC) staining

To evaluate expression of UCP1 in epididymal adipose tissue, IHC staining was performed. Epididymal adipose tissue samples were fixed in 4% paraformaldehyde for 24 hours at 4°C, followed by dehydration in a graded ethanol series and embedding in paraffin. Tissue sections (5 μm thick) were prepared using a microtome and mounted on glass slides. The sections were deparaffinized in xylene and rehydrated through a graded ethanol series. Antigen retrieval was performed by heating the sections in citrate buffer (pH 6.0) at 95°C for 20 minutes, followed by cooling to room temperature. Endogenous peroxidase activity was quenched by incubating the sections in 3% hydrogen peroxide for 10 minutes. After washing with phosphate-buffered saline (PBS), the sections were blocked with 5% bovine serum albumin (BSA) for 1 hour at room temperature to prevent non-specific binding. Subsequently, the sections were incubated with a primary antibody against UCP1 (Proteintech, Cat# 23,673–1-AP) overnight at 4°C. After washing with PBS, the sections were incubated with a Goat anti-rabbit Alexa Flour 488 (Abcam, #ab150077) for 1 hour at room temperature. The immunoreactivity was visualized using 3,3’-diaminobenzidine (DAB) as the chromogen, followed by counterstaining with haematoxylin. Stained sections were imaged using a light microscope (Nikon Eclipse E100).

### Pcr array and real-time qPCR

Epididymal adipose tissue was rapidly dissected from euthanized mice. Total RNA was extracted using the RNeasy Mini Kit (Qiagen, Cat#74104) according to the manufacturer’s protocol. RNA concentration and purity were determined using a NanoDrop spectrophotometer (OD260/280 ratio > 1.8), and integrity was verified by agarose gel electrophoresis. The cDNA was synthesized from 1 μg of total RNA using a reverse transcription RT^2^ First Strand Kit (Qiagen, Cat#330401). The resulting cDNA was diluted and stored at −20°C for subsequent analysis. A commercially available 86 Epigenetic Modifying Enzyme PCR array (mivectorbio, Shanghai, China) was used to profile the expression of key enzymes involved in DNA methylation, histone modification, and chromatin remodelling. The real-time PCR was performed on cDNA using RT^2^ SYBR Green qPCR Mastermix (Qiagen, Cat#330500) and loaded into the 384-well PCR array plate, which contained pre-designed primers for target genes and GAPDH. The PCR array was run on an ABI Q6 (Applied Biosystems Inc. USA) system under the following conditions: initial denaturation at 95°C for 10 minutes, followed by 40 cycles of 95°C for 15 seconds and 60°C for 1 minute. Relative gene expression levels were calculated using the 2^^−ΔΔCt^ method.

For real-time qPCR, reverse transcription was performed using the RevertAid First Strand cDNA Synthesis Kit (Thermo Scientific™, Cat#K1622), and PCR amplification was carried out using the 2 × Master Mix kit (Roche, Cat#04913914001). The remaining procedures for RNA extraction, amplification, and the PCR instrument used were identical to those described above for the PCR array.

### Isolation and culture of primary adipocytes from mice

Primary adipocytes were isolated from inguinal white adipose tissue (iWAT) of 3–4-week-old C57BL/6J mice. Following cervical dislocation, iWAT was excised under sterile conditions and rinsed 3–5 times with PBS containing 1% penicillin – streptomycin (Sangon, Cat# E607011) until the supernatant became clear. The tissue was minced into small fragments and digested in DMEM/F12 (Corning, Cat# 10–092-CVR) containing 1 mg/mL type II collagenase (Sigma, Cat# C6885) at 37°C in a 5% CO_2_ incubator (Thermo, FORMA SERIES II WATER JACKET) for 40 min with gentle agitation every 10 min. The digestion was terminated by adding serum-free DMEM/F12, and the suspension was sequentially filtered through 100 µm and 40 µm cell strainers (Corning, Cat# 352,360 and 352,340) to remove undigested tissue. The filtrate was centrifuged at 200 × g for 3 min. The floating mature adipocytes were discarded, and the pelleted fraction containing the stromal vascular fraction (SVF) cells was resuspended in DMEM/F12 supplemented with 10% foetal bovine serum (FBS; Gibco). The SVF cells were seeded into six-well plates (Falcon, Cat# 353,108) at a density of 1 × 10^5^ cells/cm^2^ and incubated at 37°C with 5% CO_2_ for 4 h. After attachment, nonadherent cells and debris were removed by washing 3–5 times with PBS. When SVF-derived cells reached full confluence after approximately 48 h of culture, adipogenic differentiation was induced by adding a differentiation cocktail containing 1 μM dexamethasone (Beyotime, ST1258), 250 μM 3-isobutyl-1-methylxanthine (Beyotime, ST1398), 0.5 μg/mL bovine insulin (iCell, iCell-0016-a), and 60 μM rosiglitazone (Beyotime, Y174537). After 48 h of induction, the medium was replaced with maintenance medium containing only insulin (10 μg/mL) and rosiglitazone (2.5 μM), while dexamethasone and IBMX were withdrawn. The cells were subsequently cultured for an additional 6–8 days with medium renewal every 2–3 days, until mature adipocytes were obtained for downstream analyses.

### Cell culture and transfection

3T3-L1 (Mouse embryonic fibroblasts) were purchased from iCell Bioscience Inc (#iCell-m066). 3T3-L1 preadipocytes were cultured in Dulbecco’s Modified Eagle’s Medium (DMEM, containing 1.5 g/L NaHCO_3_) (#iCell-128–0001) supplemented with 10% foetal bovine serum (FBS), 1% GlutaMAX-1 (#iCell-0900), 1% MEM non-essential amino acid (#iCell-01000), 1% sodium pyruvate (#iCell-01100), and 1% penicillin-streptomycin (#E607011, Sangon) at 37°C in a humidified atmosphere with 5% CO_2_. For adipogenic induction differentiation, two days post-confluence (designated as Day 0), cells were induced to differentiate using adipogenic induction medium (AIM) (Procell, #PD-031) consisting of DMEM supplemented with 10% FBS, 0.5 mM 3-isobutyl-1-methylxanthine (IBMX), 1 μM dexamethasone, and 10 μg/mL insulin. After 48 hours (Day 2), the medium was replaced with DMEM containing 10% FBS and 10 μg/mL insulin (adipocyte maintenance medium). The maintenance medium was replaced every 2 days until Day 8, when mature adipocytes were fully differentiated.

Overexpression of WDR4, the full-length cDNA of WDR4 was cloned into the pcDNA3.1 vector to generate the pcDNA3.1-WDR4 overexpression plasmid. The empty pcDNA3.1 vector was used as a negative control.

For BMP8B knockdown, three siRNAs targeting BMPB8 and one si-NC control were synthesized, the sequences are shown in Supplemental Table S1.

For transfection, 3T3-L1 cells or primary adipocytes were seeded in 6-well plates at a density of 2 × 10^5^ cells per well and cultured until reaching 70–80% confluency. Cells were transfected with either pcDNA3.1-WDR4 or the empty vector using Lipofectamine 2000 (Invitrogen, #1166019) according to the manufacturer’s protocol. After 24 hours, cells were treated with 5 mM 3-MA (MCE, #HY-19312) for 24 hours. Another, for bafilomycin A1 treatment, cells were treated with 100 nM bafilomycin A1 (MCE, #HY-100558) for 24 hours.

### Western blotting

Differentiated 3T3-L1 adipocytes were lysed in RIPA buffer (Thermo). The protein concentration was determined using a BCA protein assay kit (Pierce). Equal amounts of protein 20 μg were resolved by 10% SDS-PAGE, followed by transferred onto a PVDF membrane. The membrane was blocked with 5% non-fat milk in Tris-buffered saline containing 0.1% Tween-20 (TBST) for 1 hour at room temperature. Subsequently, the membrane was incubated overnight at 4°C with primary antibodies against the anti-WDR4 (1:200, #sc100894, Santa Cruz), anti-UCP1 (1:5000, 23,673–1-AP, Proteintech), anti-PGC-1α (1:1000, AF5301, Affinity), anti-PPARA (1:1000, AF5395, Affinity), anti-puromycin (1:1000, ZMS1016-25 UL, MERCK), anti-TGIF2 (1:5000, 11,522–1-AP, Proteintech), anti-BMP8B (1:1000, DF3850, Affinity), anti-AMH (1:1000, 14,461–1-AP, Proteintech), anti-GAPDH (1:15000, 60,004–1-lg, Proteintech). After three washes with TBST, the membrane was incubated with Goat Anti-Mouse IgG H&L(HRP) (1:1000, #ab205719, Abcam) or Goat Anti-Rabbit IgG H&L(HRP) (1:20000, #ab6721, Abcam) for 1 hour at room temperature. Following three additional washes with TBST, protein bands were visualized using enhanced chemiluminescence (ECL) substrate (Thermo) and imaged using a chemiluminescence detection system (Shanghai Qinxiang Scientific Instrument Co., LTD).

### Oil red O staining

Cells were fixed with 4% paraformaldehyde for 30 minutes at room temperature and washed twice with PBS. Next, cells were stained with Oil Red O solution for 30 minutes protected from light at room temperature to visualize lipid droplets. After washing cells with distilled water, the nuclei were counterstained with haematoxylin for 1–2 minutes. Finally, cells were observed under a light microscope.

### Transmission electron microscopy (TEM)

After cell digestion, the supernatant was removed by centrifugation. The cell pellet was fixed in 2.5% glutaraldehyde at 0–4°C for 1–3 hours. Following fixation, the samples were washed three times with 0.1 M phosphate buffer (10 minutes per wash). Post-fixation was performed using 1–2% osmium tetroxide for 15–40 minutes at room temperature. The fixed samples were dehydrated through a graded ethanol series: 30%, 50%, 70%, 80%, 90%, and 95% ethanol (5–10 minutes per step), followed by three changes of 100% ethanol (10–15 minutes each). After complete dehydration, the samples were infiltrated with a 1:1 mixture of 100% ethanol and epoxy resin embedding medium for 30 minutes to several hours at room temperature. Subsequently, the samples were transferred to pure epoxy resin embedding medium and incubated for 6 hours or overnight at room temperature. For embedding, the infiltrated samples were placed into pre-labelled embedding moulds filled with fresh epoxy resin. The moulds were then polymerized at 60°C for 24 hours to form solid blocks. Ultrathin sections (50–70 nm thick) were cut using a Leica Ultramicrotome (Leica Microsystems). The sections were collected on copper grids for further uranyl acetate staining for 15 minutes, followed by staining with lead citrate for 5 minutes. After air-drying, the grids were examined using a transmission electron microscope (JEM-1200EX, Nippon Electronics Corporation).

### Immunofluorescence (IF) staining

Differentiated 3T3-L1 adipocytes were seeded in 12-well plates at a density of 3 × 10^4^ cells per well and allowed to adhere overnight. After reaching 70–80% confluency, the cells were subjected to IF staining. The culture medium was aspirated, and the cells were fixed with 300 μL of 4% paraformaldehyde in PBS for 30 minutes at room temperature. After fixation, the cells were permeabilized with 300 μL of 0.3% Triton X-100 in PBS for 5 minutes. Following permeabilization, non-specific binding sites were blocked by incubating the cells with 300 μL of 3% bovine serum albumin in PBS for 30 minutes. After blocking, the cells were incubated with 200 μL of the diluted primary antibody DRP1 (Proteintech, #12957–1-AP) and LC3 (Proteintech, #14600–1-AP) per well overnight at 4°C. and then incubated with secondary antibody Goat anti-rabbit Alexa Flour 488 (Abcam, #ab150077) per well for 1 hour at room temperature. Cells were stained with 300 μL of DAPI (1 μg/mL in PBS) per well for 10 minutes at room temperature, protected from light. Finally, fluorescence images were acquired using a fluorescence microscope (Olympus, CKX53).

### tRNA reduction and cleavage sequencing (Trac-seq)

Total RNA was extracted from the differentiated 3T3-L1 adipocytes with WDR4 overexpression using RNeasy Mini Kit (Qiagen, Cat#74104) following the manufacturer’s protocol. RNA quality and concentration were assessed using a NanoDrop spectrophotometer and agarose gel electrophoresis. Small RNAs, including tRNAs, were isolated from total RNA using the mirVana™ miRNA separation Kit (#AM1561, Thermo Fisher Scientific). To remove methylations (such as m6A, m1A, and m3C) that could interfere with downstream steps, the small RNA fraction was treated with AlkB at 37°C for 2 hours. The RNA was then reduced with sodium borohydride (NaBH_4_) to convert m7G to dihydrom7G (Dhm7G), which is more susceptible to aniline-induced cleavage. The reduced RNA was treated with aniline (pH 4.5) at 60°C for 10 minutes to induce cleavage at Dhm7G sites. The reaction was stopped by ethanol precipitation. Cleaved RNA fragments were ligated to 5’ and 3’ adaptors and the adaptor-ligated RNA was reverse-transcribed into cDNA using NEBNext Multiplex Small RNA Library Prep Set for Illumina (Set 1) (#E7300S, NEB). The cDNA was amplified using PCR with forward and reverse primers complementary to the 5’ and 3’ adaptors, respectively. The PCR products were purified using AMPure XP beads (Beckman Coulter). The final library was quantified using a Qubit™RNA fluorometer (#Q32852, Thermo Fisher Scientific) and sequenced on an Illumina NovaSeq^TM^ X Plus platform with 150 bp single-end reads by Mivectorbio (Shanghai, China). The raw sequencing data were deposited in the National Genomics Data Center (NGDC) Genome Sequence Archive (GSA) under accession number PRJCA043363.

Raw reads were quality-checked using FastQC (v0.11.9) and trimmed to remove adaptor sequences and low-quality bases using Cutadapt (v3.4) or Trimmomatic (v0.39). Cleaned reads were aligned to the reference genome GRCm38/mm10 using Bowtie. tRNA sequences were annotated using GtRNAdb databases. Cleavage sites corresponding to m7G modifications were identified by locating the 5’ ends of reads that aligned to tRNA sequences. The frequency of cleavage at each site was quantified using custom scripts. MEME (https://meme-suite.org/meme/tools/meme) was applied to develop m7G sites around 7 base sequence to search motif.

### Statistical analysis

Statistical analysis was performed using GraphPad Prism 9.0 software. Data were expressed as mean ± SD, and differences between groups were analysed by Student’s t-test or one-way ANOVA followed by Tukey’s post-hoc test. A p-value < 0.05 was considered statistically significant.

## Results

### The tRNA m^7^G methyltransferase complex METTL1/WDR4 downregulated in HFD-induced obesity male mice

**Figure 1.**
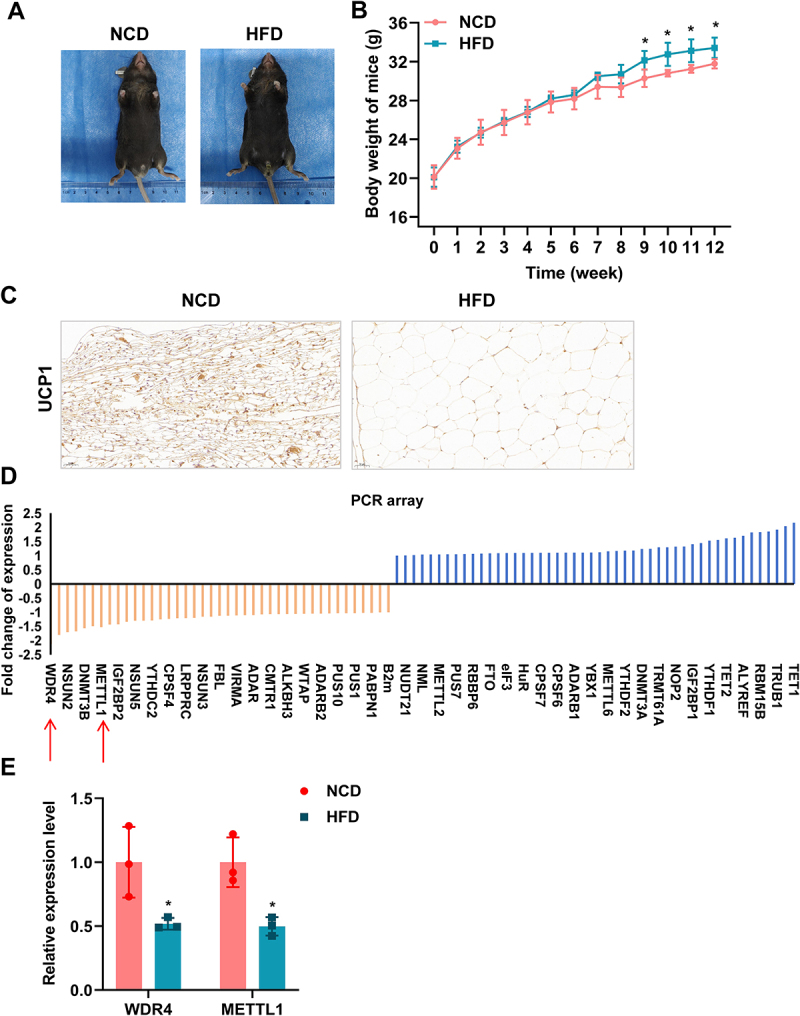
tRNA m^7^G methyltransferase complex METTL1/WDR4 downregulated in HFD-induced obesity mice. (a) Representative images of mice fed with NCD (*n* = 5) and HFD (*n* = 5). (b) a line graph depicting the changes in body weight of mice fed with NCD and HFD over time. (c) Representative IHC images showing UCP1 protein expression. (d) bar graphs illustrating the results of pcr array analysis of epididymal adipose tissue from NCD and HFD-fed mice. (e) real-time qPCR analysis of WDR4 expression in the epididymal adipose tissue of NCD and HFD-fed mice. * means *p* < 0.05.

To screen the epigenetic modifying enzymes that are associated with BAT activation, an HFD-induced obese male mouse model was established, and a PCR array on epididymal adipose tissue from mice was performed. As shown in Figures 1(A-B), compared to the NCD group, the HFD group exhibited a significant increase in body weight in mice. IHC analysis confirmed reduced expression of the obesity-related thermogenic marker UCP1 and revealed enlarged lipid droplets in epididymal adipose tissue of HFD mice, indicative of BAT dysfunction (Figure 1(C)), validating successful model establishment. Although the visual magnitude of body weight gain was relatively modest, the difference between the NCD and HFD groups was statistically significant. Moreover, previous studies report that short-term or moderate high-fat feeding rapidly provokes BAT lipid accumulation and impaired insulin responsiveness, hallmarks of BAT dysfunction, sometimes occurring before pronounced whole-body weight gain [20,21]. Therefore, our histological and IHC findings support the conclusion that HFD induced functionally relevant BAT impairment in this model.

Subsequent analysis of a Mouse Epigenetic Modification Enzymes PCR Array revealed that the expression of WDR4 showed the most pronounced downregulation in the HFD group (Figure 1(D)). Therefore, we selected WDR4 for further investigation.

The METTL1/WDR4 complex is a critical mediator of N^7^-methylguanosine (m^7^G) modification, a conserved RNA modification that plays essential roles in RNA metabolism and function [22]. METTL1, the catalytic subunit, and WDR4, the regulatory subunit, form a stable complex that catalyzes the transfer of a methyl group from S-adenosylmethionine to the N^7^ position of guanine in RNA molecules, particularly in tRNAs [23]. Real-time qPCR confirmed that the expression of METTL1 and WDR4 was significantly reduced in HFD mice compared to NCD controls (Figure 1(E)). In all, the tRNA m^7^G methyltransferase complex protein METTL1/WDR4 were downregulated in HFD-induced obesity mice.

### WDR4 promotes adipocyte browning through mitophagy

**Figure 2.**
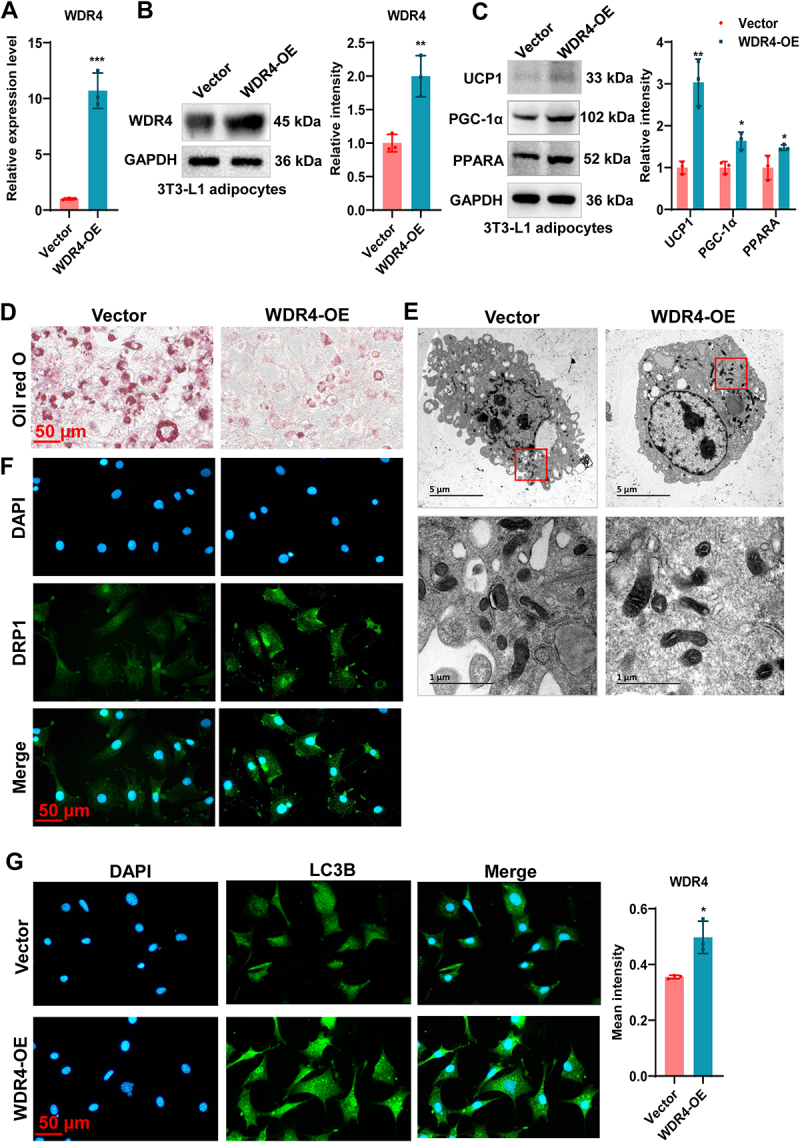
WDR4 promotes adipocyte browning and mitophagy. (a) the overexpression efficiency of WDR4 was detected by real-time qPCR in 3T3-L1 adipocytes. (b) the overexpression efficiency of WDR4 was detected by western blot. (c) western blot analysis of UCP1, PGC-1α, and PPARA expression in 3T3-L1 adipocytes. (d) lipid droplet was stained by oil red O staining. (e) mitophagy of WDR4-overexpressing 3T3-L1 adipocytes was observed by tem. The expression of DRP1 (f) and LC3 (g) was detected by IF staining in WDR4-overexpressing 3T3-L1 adipocytes. ** means *p* < 0.01, *** means *p* < 0.001.

To explore the effect of WDR4 on BAT activation, 3T3-L1 cells were transfected with the pcDNA3.1-WDR4 overexpression plasmid on day 6 of adipogenic differentiation induced by AIM. The overexpression efficiency of WDR4 was confirmed by both real-time qPCR and western blot (Figure 2(A-B)). Importantly, overexpression of WDR4 in 3T3-L1 adipocytes enhanced the protein expression of browning markers, including UCP1, PGC-1α, and PPARA (Figure 2(C)) and suppressed lipid droplet formation as evidenced by oil red O staining (Figure 2(D)). These findings suggest that WDR4 promotes adipocyte browning and may play a protective role against obesity.

Considering that UCP1-mediated browning of white adipocytes is achieved through enhanced mitochondrial quantity and function [3], coupled with the established importance of mitochondrial homoeostasis in modulating adipose tissue browning [15], and given the fundamental role of mitophagy in maintaining mitochondrial quality control and cellular metabolic regulation, we therefore prioritized investigating mitophagy in our subsequent studies. TEM was employed to visualize mitochondrial ultrastructure and autophagic vesicles. In the control group, mitochondria exhibited a normal morphology with intact cristae and minimal autophagic activity. In contrast, WDR4-overexpressing 3T3-L1 cells displayed a significant increase in autophagic vesicles engulfing mitochondria, indicative of enhanced mitophagy (Figure 2(E)). Furthermore, we performed IF staining to detect the expression of key mitophagy markers, DRP1 and LC3. DRP1, a mediator of mitochondrial fission, was significantly upregulated in WDR4-overexpressing cells compared to the control group (Figure 2(F)). Similarly, the punctate staining pattern of LC3, a marker of autophagosome formation, was more pronounced in WDR4-overexpressing cells, indicating increased autophagic flux (Figure 2(G)). These results suggest that WDR4 promotes adipocyte browning through enhanced mitophagy.

**Figure 3.**
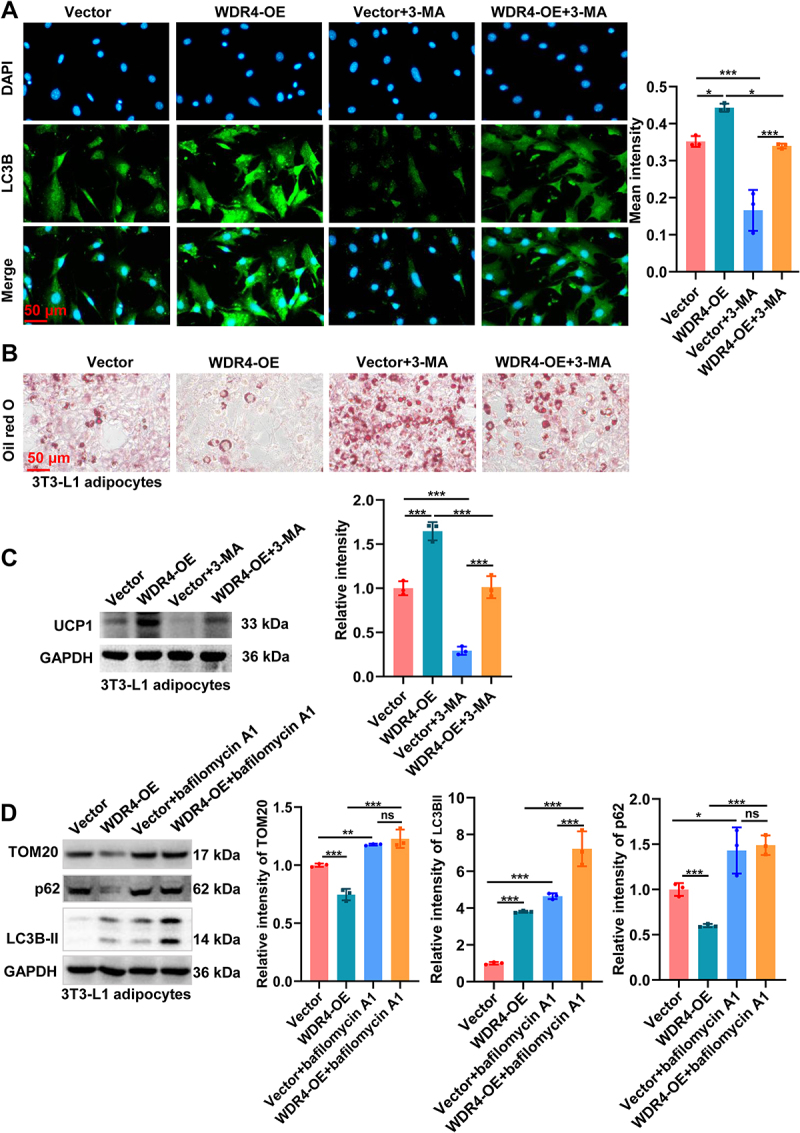
WDR4-mediated mitophagy is essential for adipocyte browning. (a) the expression of LC3 was detected by IFstaining in WDR4-overexpressing 3T3-L1 adipocytes with 3-ma treatment, scale bar: 50 μm. (b) lipid droplet was stained by oil red O staining (scale bar: 50 μm). (c) western blot analysis of UCP1 expression in 3T3-L1 adipocytes with 3-ma treatment. (d) western blot analysis of LC3-I/II, p62 and TOM20 in four groups: vector, WDR4-OE, vector + bafilomycin A1, and WDR4-OE + bafilomycin A1. * means *p* < 0.05, ** means *p* < 0.01, *** means *p* < 0.001.

To further determine whether WDR4-mediated mitophagy is essential for adipocyte browning, we treated WDR4-overexpressing differentiated 3T3-L1 adipocytes with 3-MA, a mitophagy inhibitor. The enhanced expression of LC3 induced by WDR4 overexpression was obviously attenuated by 3-MA (Figure 3(A)), indicating mitophagy blocking. Importantly, inhibition of mitophagy using 3-MA significantly abolished the browning effects of WDR4 overexpression, as evidenced by reduced UCP1 expression level and lipid droplet formation (Figure 3(B-C)). To further confirm this observation, we performed additional experiments using bafilomycin A1, an inhibitor that blocks the fusion of autophagosomes and lysosomes. Western blot analysis showed that WDR4 overexpression markedly increased LC3-II levels, accompanied by reduced p62 and TOM20 expression, consistent with increased autophagosome formation and selective removal of mitochondria, suggesting enhanced mitophagic activity. However, bafilomycin A1 treatment led to significant accumulation of LC3-II and p62 and prevented the WDR4-induced decrease of TOM20, resulting in higher TOM20 levels in bafilomycin-treated groups (Figure 3(D)). Collectively, these data indicate that WDR4 facilitates adipocyte browning by activating mitophagy, and that blockade of autophagic flux by either 3-MA or bafilomycin A1 abrogates WDR4-mediated mitophagic degradation of mitochondrial proteins.

**Figure 4.**
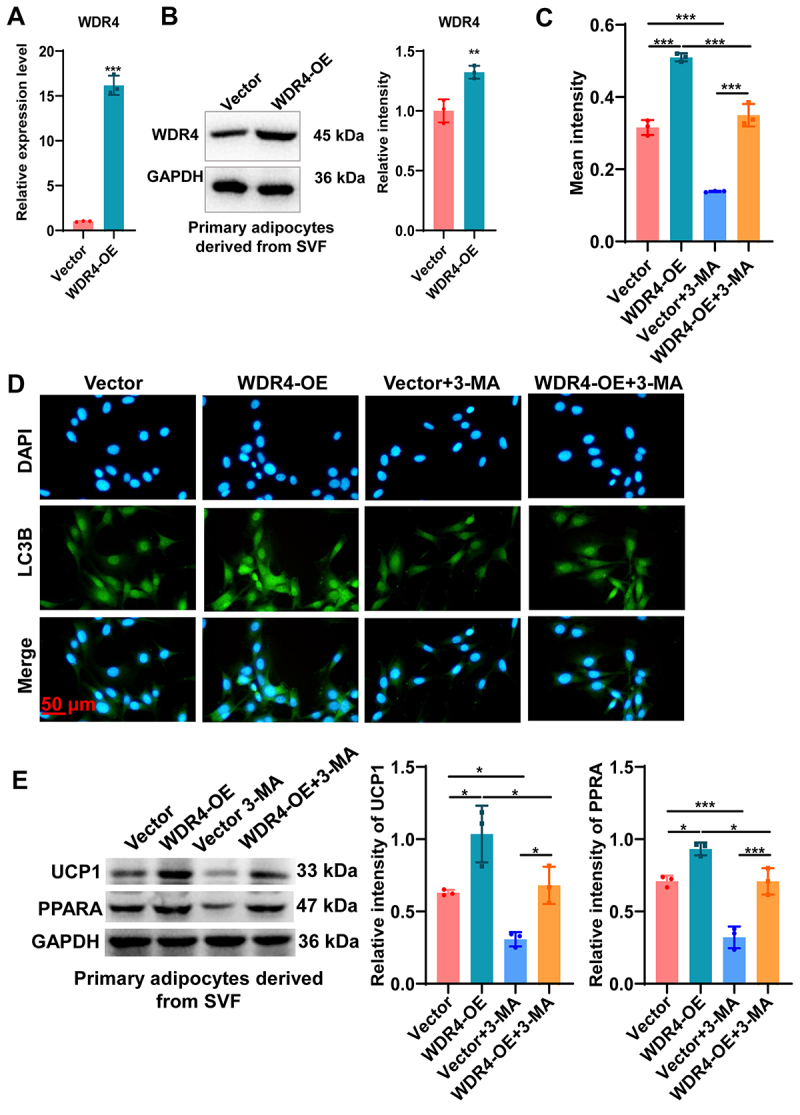
WDR4 promotes adipocyte browning through autophagy activation in primary SVF-derived adipocytes. (a, b) RT-qPCR and western blot analyses confirming the efficiency of WDR4 overexpression in SVF-derived primary adipocytes from mouse inguinal white adipose tissue (iWAT). (c, d) immunofluorescence staining and quantification of LC3B puncta showing increased autophagosome formation upon WDR4 overexpression. Treatment with the autophagy inhibitor 3-ma significantly reduced LC3B signal intensity. Scale bar, 50 μm. (e) western blot analysis of UCP1 and PPARA expression in the four groups. *p < 0.05, **p < 0.01, ***p < 0.001.

To validate findings obtained in 3T3-L1 adipocytes, we isolated primary SVF-derived adipocytes from mouse iWAT and overexpressed WDR4. The efficiency of WDR4 overexpression was confirmed by RT-qPCR and western blot analyses (Figure 4(A-B)). Compared with control cells, WDR4-overexpressing primary adipocytes exhibited significantly increased LC3 puncta formation, as shown by LC3B immunofluorescence (Figure 4(C-D)). In contrast, treatment with the autophagy inhibitor 3-MA substantially reduced LC3B signal intensity and abolished WDR4-induced autophagic activation. Consistent with these findings, western blot analysis demonstrated that WDR4 overexpression upregulated key browning markers UCP1 and PPARA, while 3-MA treatment reversed these effects in primary adipocytes (Figure 4(E)). Together, these results confirm that WDR4 promotes adipocyte browning in primary adipocytes through activation of autophagy.

### m^7^G Trac-seq profiled tRNA m^7^G modification at single-nucleotide resolution following WDR4 overexpression

**Figure 5.**
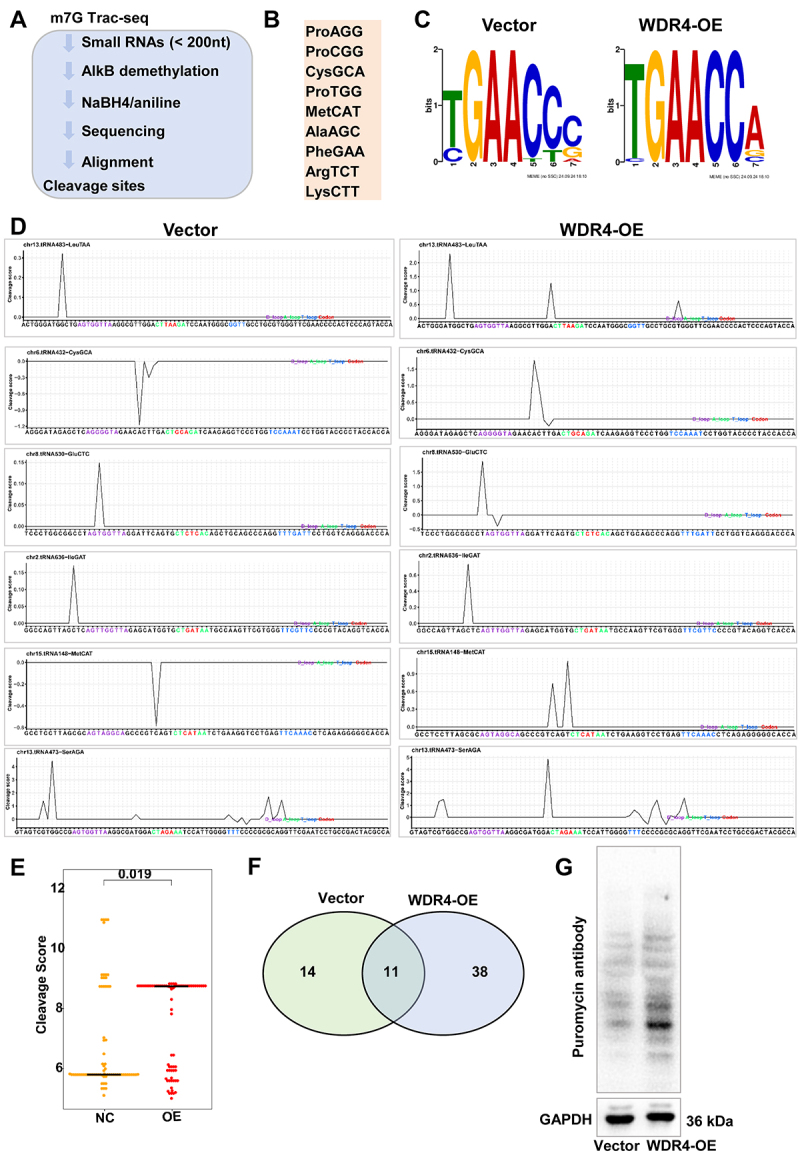
m^7^G Trac-seq profiled tRNA m^7^G modification upon on the WDR4 overexpression. (a) a schematic diagram illustrating the workflow of m^7^G Trac-seq based on the chemical cleavage method. (b) list of m^7^G-modified tRnas identified by Trac-seq in WDR4-overexpressing 3T3-L1 adipocytes. (c) motif sequence at m^7^G site. (d) Representative images of peaks of m^7^G-modified tRnas. (e) quantification of m^7^G score on m^7^G-modified tRnas. (f) a venn diagram depicting the common and unique m^7^G-modified tRnas identified in control and WDR4-overexpressing 3T3-L1 adipocytes. (g) puromycin incorporation assay in 3T3-L1 adipocytes. Western blot detection of puromycin-labeled nascent polypeptides indicates enhanced translational activity in the WDR4-overexpressing group compared with the vector control. Cells were treated with 10 µg/mL puromycin for 20 min before harvest.

To comprehensively characterize the tRNA m^7^G methylome regulated by WDR4 in 3T3-L1 adipocytes, we employed a chemical cleavage-based approach coupled with high-throughput sequencing (m^7^G Trac-seq) to map m^7^G modifications at single-nucleotide resolution (Figure 5(A)). Our analysis identified a total of 9 tRNAs harbouring m^7^G modifications in two groups (Figure 5(B)). Cleavage site profiling revealed a conserved ‘TGAACCx’ motif associated with m^7^G modification (Figure 5(C)). Notably, WDR4 overexpression significantly increased the abundance of m^7^G modifications across multiple tRNAs (Figure 5(D,E)). For instance, the cleavage scores of tRNA^LeuTAA^, tRNA^CysGCA^, tRNA^GluCTC^, tRNA^IleGAT^, tRNA^MetCAT^, and tRNA^SerAGA^ were markedly elevated in the WDR4-overexpressing group compared to the control (Figure 5(D)). Furthermore, WDR4 overexpression induced 38 unique tRNA m^7^G modification sites, highlighting its role as a key regulator of tRNA m^7^G methylation (Figure 5(F)). These findings demonstrate that WDR4 overexpression reshapes the tRNA m^7^G methylome in 3T3-L1 adipocytes, enhancing m^7^G modification at specific tRNA sites.

### WDR4-mediated tRNA m^7^G modification modulates BMP8B translation

**Figure 6.**
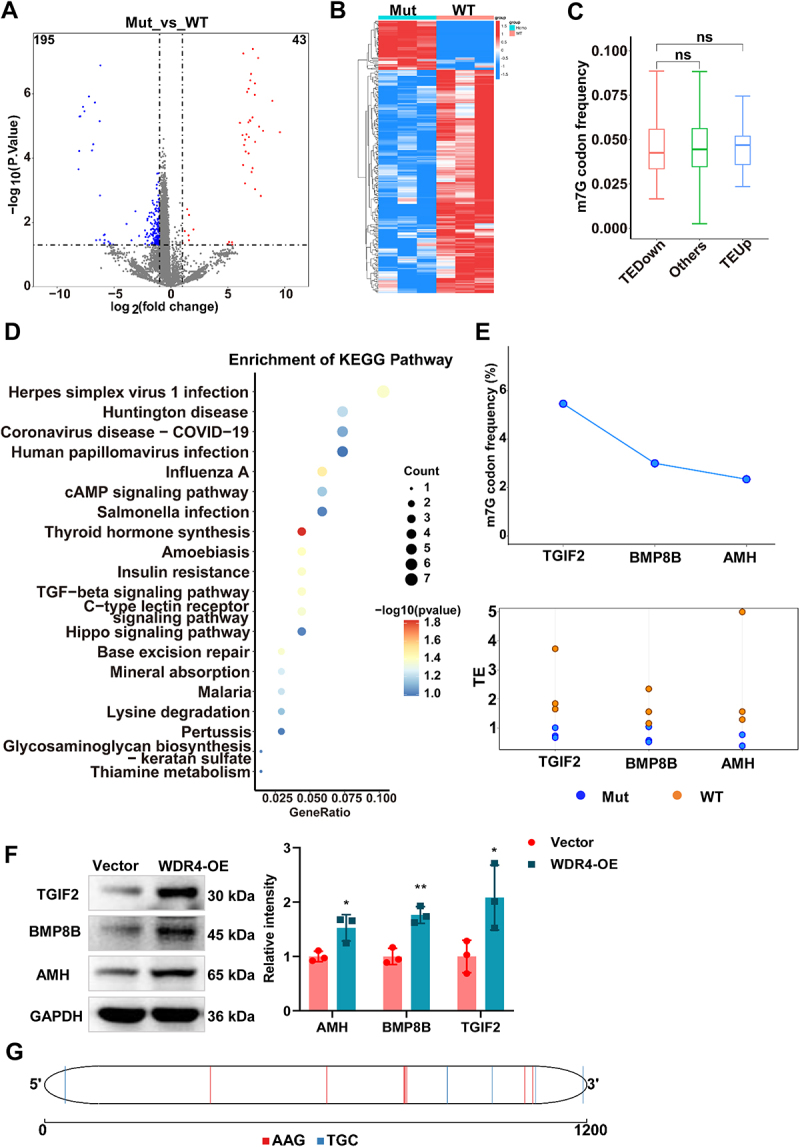
WDR4 modulates BMP8B translation via tRNA m7G modification. (a) a volcano plot illustrating the mRNAs with differential te between WDR4-mutant and wild-type 3T3-L1 adipocytes. (b) a heat map of the mRNAs with differential translation efficiency between WDR4-mutant and wild-type 3T3-L1 adipocytes. (c) frequencies of m^7^G-related codons in TE-increased genes (TE-up), TE-decreased genes (TE-down), and other genes (others) in WDR4-overexpressing 3T3-L1 adipocytes. (d) KEGG enrichment of TE-decreased genes. (e) frequency analysis of the m^7^G-modified tRnas decoded codons in 3 mRNAs enriched in TGF-β signaling pathway. (f) western blot analysis of TGIF2, amh, and BMP8B expression in WDR4-overexpressing 3T3-L1 adipocytes. (g) the distribution of codons AAG and TGC on the BMP8B gene.

**Figure 7.**
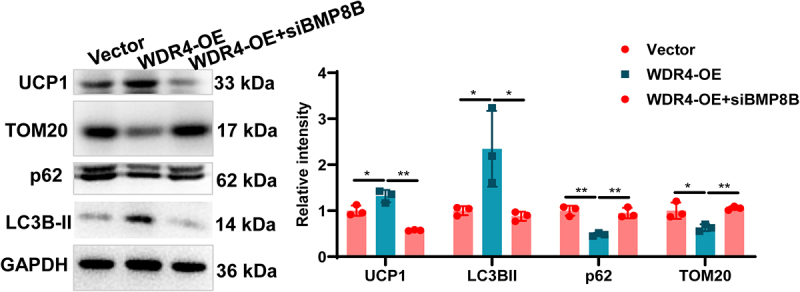
BMP8B mediates WDR4-induced mitophagy and adipocyte browning. western blot analysis of LC3-I/II, p62, TOM20, and UCP1 in 3T3-L1 adipocytes transduced with vector, WDR4-overexpression, or WDR4-OE combined with BMP8B knockdown. *p < 0.05, **p < 0.01, ***p < 0.001.

Given the critical role of tRNAs in mRNA translation, we next investigated the impact of WDR4 on mRNA translation efficiency (TE) in 3T3-L1 adipocytes. To this end, we performed a puromycin incorporation assay to assess global protein synthesis activity. Western blot analysis showed that puromycin-labelled nascent polypeptides were markedly increased in WDR4-overexpressing adipocytes compared with control cells, indicating enhanced translational activity upon WDR4 overexpression (Figure 5(G)). To further substantiate this observation, we analysed the GSE229240 dataset from the GEO database, which includes ribosome profiling (RNC-seq) and RNA-seq data from both WDR4-mutant and wild-type (WT) samples. Using the limma package, we identified genes with differential TE between the WDR4-mutant and WT groups, applying a threshold of |log2FC| > 1 and P-value < 0.05. Our analysis revealed that the WDR4 mutation significantly altered the TE of 224 genes, with 43 genes showing increased TE and 195 genes exhibiting decreased TE (Figures 6(A-B)). Since m^7^G modification is known to enhance tRNA-mRNA pairing efficiency and thereby promote translation [24], we further explored the relationship between m^7^G-modified tRNAs and differentially translated mRNAs. Specifically, we calculated the number of codons in the differentially translated mRNAs that are decoded by the 38 m^7^G-modified tRNAs uniquely identified in the WDR4-overexpression group (Figure 5(F)). The results demonstrated that mRNAs with enhanced TE had a higher number of codons decoded by m^7^G-modified tRNAs (Figure 6(C)). To gain functional insights into the 195 genes with reduced TE upon WDR4 mutation, we performed KEGG pathway enrichment analysis. These genes were significantly enriched in several signalling pathways, including TGF-β signalling pathway which emphasized by TGIF2, AMH, and BMP8B (Figure 6(D)). Notably, the TGF-β pathway promotes mitochondrial translocation and activates mitophagy [25]. Within the TGF-β pathway, BMP8B, a gene known to enhance thermogenesis in brown adipose tissue through both central and peripheral mechanisms [26], and BMP8B and TGIF2 exhibited higher TE and a greater abundance of m7G-related codons (Figure 6(E)). Western blot also confirmed the enhanced TE of TGIF2, AMH, and BMP8B in the 3T3-L1 cells after WDR4 overexpression (Figure 6(F)). BMP8B was of particular interest, and the distribution of codons AAG and TGC on the BMP8B gene is illustrated in Figure 6(G). We wonder the potential role of BMP8B in mediating WDR4-driven mitophagy and adipocyte browning, thus, we silenced BMP8B in WDR4-overexpressing 3T3-L1 cells. Western blot analysis revealed that WDR4 overexpression alone enhanced mitophagy and browning, while knockdown of BMP8B in WDR4-overexpressing cells partially reversed the effects, as reflected by reduced LC3-II, restoration of p62 and TOM20, and decreased UCP1 expression (Figure 7(A)). In summary, our findings indicate that WDR4 regulates mRNA translation efficiency through tRNA m^7^G modification to enhance BMP8B expression, thereby boosting mitophagy and adipocyte browning.

## Discussion

**Figure 8.**
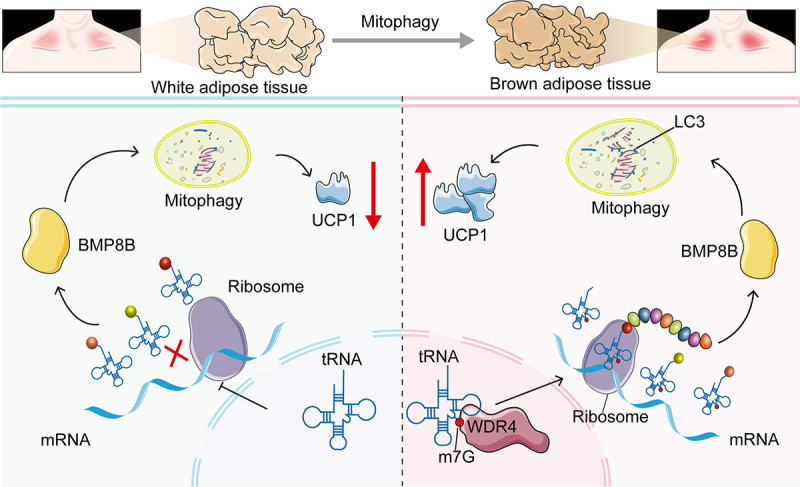
Schematic diagram of mechanism. WDR4 drives mitophagy through enhancing BMP8B expression by increasing its translation efficiency by tRNA m^7^G modification, thereby promoting adipocyte browning and improving metabolic health in the context of obesity.

Unlike white adipose tissue, brown adipose tissue is characterized by its high mitochondrial content and unique ability to dissipate energy as heat through fat oxidation, a process known as non-shivering thermogenesis [27]. BAT is predominantly abundant in neonates; however, both its quantity and activity decline with age [28]. Recent studies have revealed that BAT is also present in adults, and its activity is inversely correlated with obesity. Activation or expansion of BAT can enhance basal metabolic rate, thereby reducing fat accumulation and restricting obesity [5]. It should be noted, however, that while BAT activation and adipocyte browning show clear metabolic benefits in rodent models, translation to human obesity therapy has been challenging. Human trials using browning strategies, particularly β3-adrenergic agonists, have generally failed to induce substantial weight loss [29–31]. Nevertheless, our study provides important mechanistic insights into the regulation of BAT and adipocyte browning, which may guide future translational studies and therapeutic development. Our findings demonstrated that WDR4 drives mitophagy through enhancing BMP8B expression by increasing its translation efficiency by tRNA m^7^G modification, thereby promoting adipocyte browning and improving metabolic health in the context of obesity (Figure 8). These results highlight the critical role of WDR4-mediated epitranscriptomic regulation in linking mitophagy to adipose tissue metabolism, offering novel insights into the molecular mechanisms of adipocyte browning and BAT activation, while recognizing that direct translation to human obesity therapy requires further investigation.

This study reveals that WDR4 was downregulated in the context of obesity, and overexpression of WDR4 induced brown fat activation by enhancing mitophagy, highlighting its critical role in regulating mitochondrial homoeostasis and adipose tissue metabolism. WDR4, a co-factor of METTL1 [12], plays a crucial role in facilitating the addition of m^7^G modifications at position 46 of the tRNA variable loop, which is essential for tRNA stability and function [32]. Knockdown of WDR4 downregulates both mRNA expression and protein levels of METTL1, thereby indirectly disrupting the formation of the WDR4-METTL1 complex and reducing tRNA m^7^G modification levels [33]. Structural and biochemical analyses further reveal that WDR4 enhances the catalytic efficiency of the METTL1-WDR4 complex by promoting S-adenosylmethionine recognition and directly participating in substrate RNA binding, ensuring proper tRNA docking for effective catalysis [34]. Recent studies have demonstrated that deficiencies in METTL1 or WDR4 abolish tRNA m^7^G modifications, leading to various pathological conditions, including impaired self-renewal and differentiation of embryonic stem cells [35], microcephalic primordial dwarfism [36], and Galloway-Mowat syndrome [37]. However, the regulatory role and underlying mechanisms of WDR4 in obesity remain unexplored. This study revealed that WDR4-mediated m^7^G modification is involved in BAT activation, providing novel insights into the role of epitranscriptomic regulation in adipose tissue metabolism and obesity.

BMP8B, a member of the bone morphogenetic protein family, has emerged as a critical regulator of adipose tissue metabolism. In the context of obesity, BMP8B has been shown to play a protective role by enhancing BAT activity, for example, BMP8B knockout mice exhibit reduced thermogenesis and increased susceptibility to diet-induced obesity, whereas central BMP8B treatment enhanced sympathetic activation of BAT [26]. Interestingly, BMP8B could suppress pre-adipocytes 3T3-L1 differentiated into mature adipocytes through SMAD2/3 and NF-κB signals [38,39]. Therefore, BMP8B acts as a thermogenic protein to coordinate energy homoeostasis, a process that is particularly relevant in BAT. However, BMP8B-mediated mitophagy supports thermogenic capacity and energy expenditure currently lacks evidence in the literature. Our findings firstly demonstrate that WDR4 overexpression upregulates BMP8B expression, thereby enhancing mitophagy and promoting adipocyte browning.

Our study further supports the notion that WDR4 overexpression upregulates BMP8B expression in an m^7^G modification-dependent manner. The regulation of BMP8B by tRNA m^7^G modification represents a novel layer of epitranscriptomic control in adipose tissue metabolism. While the role of m^7^G modification in tRNA stability and translation efficiency is well-established [32,40], its impact on specific downstream targets, such as BMP8B, remains underexplored. Our findings suggest that WDR4-mediated m^7^G modification enhances BMP8B translation, thereby linking tRNA epitranscriptomics to metabolism and adipocyte browning. This is consistent with recent studies showing that m^7^G modifications can modulate the translation and metabolic reprogramming to govern ketogenesis [41]. However, further research is needed to elucidate the precise mechanisms by which m^7^G modifications influence BMP8B expression and function.

In conclusion, our study highlights the role of WDR4-mediated m^7^ G modification in enhancing BMP8B expression, thereby promoting mitophagy and adipocyte browning. These findings not only deepen our understanding of the molecular mechanisms underlying BAT activation but also open new avenues for therapeutic interventions by providing mechanistic insights that may inform future translational studies.

## Supplementary Material

Supplemental Table 1.docx

## Data Availability

The raw sequencing data were deposited in the National Genomics Data Center (NGDC) Genome Sequence Archive (GSA) under accession number PRJCA043363. The other raw data were deposited in the Figshare with DOI: 10.6084/m9.figshare.29616605.
