# Supplementary material for: WDR4-mediate tRNA m7G modification to promote mitophagy and browning of white adipose tissue for ameliorating obesity in male mice
Source: Adipocyte. 2025 Nov 25;14(1):2588888. doi: 10.1080/21623945.2025.2588888 (PMC12667666; doi:10.1080/21623945.2025.2588888)
Supplement: Supplemental Table 1.docx [file KADI_A_2588888_SM1926.docx]

Supplemental Table 1. The sequence of primers.

| Gene | Sequence (5' to 3') | Length (bp) |
| --- | --- | --- |
| m-GAPDH-F | CAAAATGGTGAAGGTCGGTGT | 118 |
| m-GAPDH-R | GAGGTCAATGAAGGGGTCGTT |  |
| mmu-WDR4-F | GGATGGCACCCTGAGACTCT | 171 |
| mmu-WDR4-R | GGAACGCACTCACACAGAAG |  |
| mmu-METTL1-F | CAGACCACACACTGCGCTA | 114 |
| mmu-METTL1-R | CATCCTTTGGATCATCATGGCTC |  |
| BMP8B-siRNA-431 | CGGAGUUCCGGAUUUACAATT  UUGUAAAUCCGGAACUCCGTT |  |
| BMP8B-siRNA-585 | GGUGCUGGAUGUCACAGCATT  UGCUGUGACAUCCAGCACCTT |  |
| BMP8B-siRNA-650 | GCCUCUAUGUGGAGACUGATT  UCAGUCUCCACAUAGAGGCTT |  |
| siRNA-NC | UUCUCCGAACGUGUCACGUTT  ACGUGACACGUUCGGAGAATT |  |
